# X chromosome-wide identification of SNVs in microRNA genes and non-obstructive azoospermia risk in Han Chinese population

**DOI:** 10.18632/oncotarget.8759

**Published:** 2016-04-16

**Authors:** Juan Ji, Yufeng Qin, Ran Zhou, Rujin Zang, Zhenyao Huang, Yan Zhang, Minjian Chen, Wei Wu, Ling Song, Xiufeng Ling, Hongbing Shen, Zhibin Hu, Yankai Xia, Chuncheng Lu, Xinru Wang

**Affiliations:** ^1^ State Key Laboratory of Reproductive Medicine, Institute of Toxicology, Nanjing Medical University, Nanjing 210029, China; ^2^ Key Laboratory of Modern Toxicology of Ministry of Education, School of Public Health, Nanjing Medical University, Nanjing 210029, China; ^3^ Epigenetics & Stem Cell Biology Laboratory, National Institute of Environmental Health Sciences, Research Triangle Park, NC 27709, USA; ^4^ Department of Children Health Care, Nanjing Maternity and Child Health Care Hospital Affiliated to Nanjing Medical University, Nanjing 210029, China; ^5^ Department of Pediatric Surgery, State Key Laboratory of Reproductive Medicine, Nanjing Children's Hospital Affiliated Nanjing Medical University, Nanjing 210008, China; ^6^ Department of Epidemiology and Biostatistics and Key Laboratory of Modern Toxicology of Ministry of Education, School of Public Health, Nanjing Medical University, Nanjing 211166, China

**Keywords:** X-linked miRNAs, non-obstructive azoospermia

## Abstract

Human X chromosome has higher densities of microRNAs (miRNAs) compared to the average densities on autosomes. Given that numbers of X-linked miRNAs can escape from meiotic sex chromosome inactivation (MSCI) silencing, it is proposed that X-linked miRNAs may play critical roles in the process of spermatogenesis. To test the hypothesis, we performed DNA capture sequencing of human X-linked miRNAs, which was followed by a two-stage case-control study to identify the non-obstructive azoospermia (NOA) related single nucleotide variants (SNVs) in 1107 NOA cases and 1191 fertile healthy controls. Eventually, we found rs5951785, located near hsa-miRNA-506/507, increased the risk of NOA, while rs1447393, near hsa-miRNA-510, decreased the risk of NOA. Functional analysis revealed that rs5951785 significantly inhibited cell proliferation and induced cell apoptosis. Taken together, our results demonstrated that X-linked miRNAs played important roles in the pathogenesis of NOA.

## INTRODUCTION

MicroRNAs (miRNAs), a novel class of small non-coding regulatory RNAs [1], can repress target gene expression through binding to the 3′ untranslated regions (3′ UTR) of mRNA [2, 3], and their dysregulation contributes to the aetiology of numerous diseases, such as cancer, metabolic diseases and viral pathogenesis [4]. Accumulating evidence demonstrates that miRNAs take part in the process of spermatogenesis [5].

Comparing to autosomes, human X chromosome, containing genes that are uniquely expressed in male spermatogenesis, has higher density of miRNAs (referred as X-linked miRNAs) [6] and 38% of them are testis-specific or testis-enriched [7]. They participate in a number of biological processes, including cellular differentiation, proliferation and apoptosis [8]. Due to the existence of the meiotic sex chromosome inactivation (MSCI), X chromosome is silenced during spermatogenesis [9]. However, some X-linked miRNAs can escape from MSCI and regulate gene expression during late meiosis and early post-meiotic stage of spermatogenesis [7, 10]. Recently, Royo et al. reported that X-linked miRNAs are expressed before prophase I but absent during pachynema [11], and X-linked miRNAs have been reported as potential contributor to the regulation of cell cycle and spermatogonial stem cell renewal, which is crucial for the regulation of spermatogenesis, especially the mitosis of spermatogonia and meiosis of spermatocytes [12].

Single nucleotide variations (SNVs) (including mutation, single nucleotide polymorphism (SNP) and insertion-deletion (INDEL)) in pre-miRNAs, flanking regions and miRNA binding regions have been demonstrated to affect miRNA-mediated regulatory function and related with human diseases (e.g. male infertility) [13]. Zhang et al. reported that SNPs in miR-1302-binding site of CGA increased the risk of idiopathic male infertility [13]. Our previous studies also demonstrated that genetic variants in miRNA biogenesis genes (*DICER1* and *DROSHA*) increased the risk of idiopathic male infertility [14].

To systematically analyze X-linked miRNA-related SNVs and their effects on non-obstructive azoospermia (NOA), the most severe form of male infertility, we performed a chromosome-wide scan for SNVs near pre-miRNAs in X chromosome followed by SNPscan and SNaPshot in two independent validation phases. Eventually, two SNPs (rs5951785 and rs1447393), located near the regions of hsa-miR-506/507 and hsa-miR-510, were found to be associated with NOA, and *in vitro* analysis was performed to clarify their potential functions in spermatogenesis.

## RESULTS

### Identifying X-linked miRNAs' SNVs by sequencing

Based on the miRNASNP (http://www.bioguo.org/miRNASNP/), customer designed arrays were used to capture the X-linked miRNAs regions including upstream (1KB) and downstream (1KB) in 96 NOA cases and 96 fertile controls. Totally, 91 regions were captured followed by high-throughput sequencing on Illumina HiSeq 2000 to generate 100 pair-end reads. On average, each sample was sequenced to an average depth of 115×, with nearly 90% of the targeted regions covered by ≥2×. Totally, we identified 139 SNVs (including one SNP with MAF<0.05 and 138 SNPs with MAF>0.05) in X-linked miRNAs regions, among which, 22 SNPs were associated with NOA risk (Supplementary Material, Table S1).

### Two-stage validation in large cohort

For fast track replication, 22 signals were included in Stage I validation using an independent Chinese population (Supplementary Material, Table S2) by custom designed SNPscan. Unexpectedly, 18 markers identified in screening stage were not observed to be associated with NOA risk in Stage I with P values > 0.05 (Supplementary Material, Table S2). Only rs547043 near hsa-miR-4330, rs5951785 near hsa-mir-506/507, rs1447393 near hsa-mir-510, and rs5985440 near hsa-miR-652 were retained associated with NOA, among which rs547043 near hsa-miR-4330 was inconsistent with screening stage. To confirm the relationship between the other 3 SNPs and NOA risk, we carried out stage II validation in another large population (Supplementary Material, Table S3). Rs5951785 and rs1447393 were both observed to be still associated with NOA in the same direction as illustrated in the screening stage and validation stage I. Next, we performed a meta-analysis of the genotype data from stage I and II. In the combined analysis, we found that rs5951785 significantly increased the risk of NOA in the Han Chinese population (*P* meta = 1.01×10^−3^, OR = 1.45, 95% CI = 1.16-1.81). Rs1447393 acted as potential protective factor on NOA (*P* meta =1.31×10^−4^, OR=0.58, 95%CI=0.44-0.77) (Table 1). To further extend our analyses, we searched the GTEx database to see whether these two variants were quantitative trait loci (eQTL) variants, albert we did not find significant eQTLs in the available data sets [15].

**Table 1:** Two SNPs in human X-linked miRNAs were identified associated with NOA and validated in two independent cohorts

MiRNA	ACC	Location in X chr. (hg19)	SNP^a^	Study	Cases^a^	Controls^a^	OR (95%CI)^b^	*P*^b^	Q^c^
hsa-mir-506/507	MI0003193	146312238-146312361	rs5951785 A > G	Validation I	423/113	471/85	1.48(1.09-2.02)	1.31×10^−2^	
				Validation II	367/99	448/85	1.42(1.03-1.96)	3.16×10^−2^	
				Combined^d^	790/212	919/170	1.45(1.16-1.81)	1.01×10^−3^	0.856
hsa-mir-510	MI0003197	146353853-146353926	rs1447393 C > G	Validation I	462/47	469/89	0.54(0.37-0.78)	1.15×10^−3^	
				Validation II	423/37	471/65	0.63(0.41-0.97)	3.49×10^−2^	
				Combined^d^	885/84	940/154	0.58(0.44-0.77)	1.31×10^4^	0.596

Chr, chromosome; ACC, Accession number in miRbase;NOA, non-obstructive azoospermia; OR, odds ratio. Combined *P* values with a fixed effect model are presented.

a Major allele/minor allele.

b OR and P values were calculated by additive model.

c *P* value for Cochrane Q statistics test.

d Combined *P* values with a fixed effect model are presented.

### Effects of SNPs on MiRNAs and their targets

**Figure 1:**
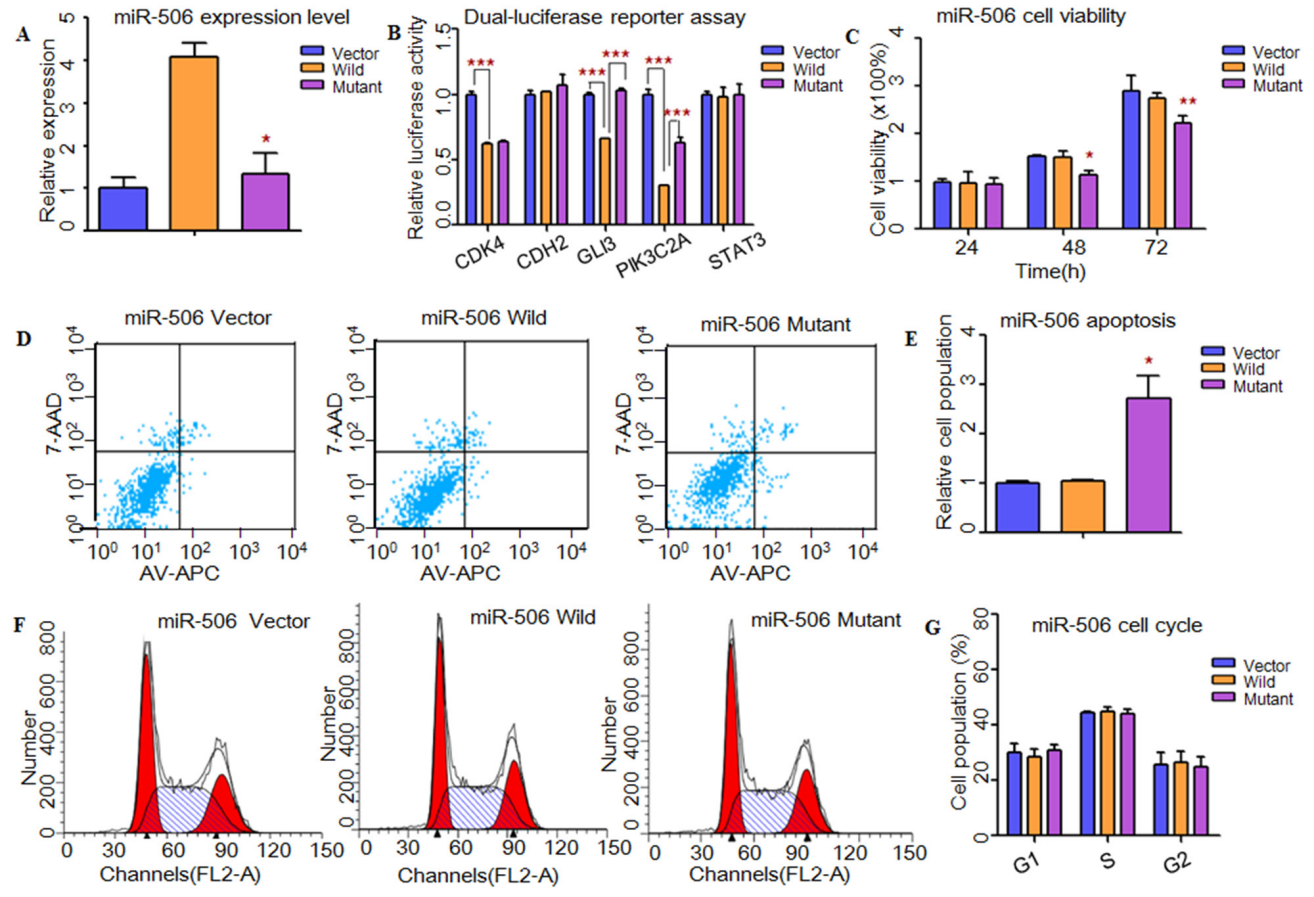
Functional analysis revealed that rs5951785 near miR-506 might contribute to the risk of NOA **A.** Quantitative real-time PCR was applied to detected mature miR-506 expression levels in transfected HEK-293T cells. The miR-506 expression level was significant down-regulated in mutant type compared with the wild type. **B.** Dual-luciferase reporter assay was conducted to measure effects of rs5951785 near miR-506 on promoter-transcriptional activity, it was increased in HEK-293T cells with miR-506 mutant type for *GLI3* and *PIK3C2A*. **C.** CCK8 was used to determine the influence of rs5951785 near miR-506 on cell growth. Rs5951785 near miR-506 significantly inhibited the HEK-293T cell growth at 48h and 72h. **D, E.** Flow cytometry assay was conducted to measure the effects of rs5951785 near miR-506 on cell apoptosis. (E) Histogram of cell apoptosis was presented to depict cell apoptotic percentages. The cell apoptosis population was markedly increased in HEK-293T cells transfected with miR-506 mutant type. **F, G.** Effects of rs5951785 near miR-506 on cell cycle were performed with flow cytometry. (G) Results quantitated in cell cycle were shown in histogram. There was no difference of cell cycle between miR-506 wild and miR-506 mutant type. Each data point represented the mean ± SE from three separate experiments in which treatments were performed in triplicate. *P<0.05, **P<0.01, ***P<0.001.

**Figure 2:**
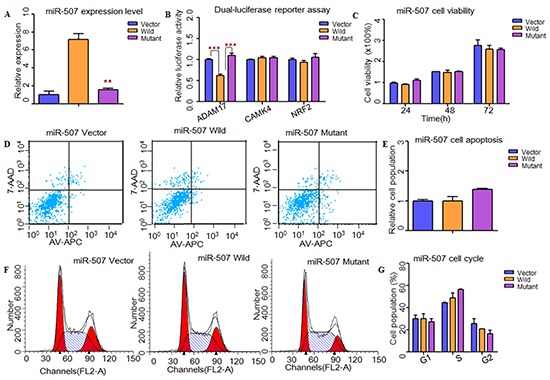
Functional analysis demonstrated that rs5951785 near miR-507 might contribute to the risk of NOA **A.** RT-PCR was applied to detected mature miR-507 expression levels. The miR-507 expression level was significant down-regulated in mutant type compared with the wild type. **B.** Dual-luciferase reporter assay was conducted to measure the effects of rs5951785 near miR-507 on promoter-transcriptional activity. It was significantly increased in miR-507 mutant type for *ADAM17*. **C.** CCK8 was used to determine the influence of rs5951785 near miR-507 on cell growth. No significant difference was observed in cell proliferation of the rs5951785 near miR-507. **D, E.** Flow cytometry assay was conducted to measure the effects of rs5951785 near miR-507 on cell apoptosis. (E) Histogram of cell apoptosis was presented to depict cell apoptotic percentages. There was no significant difference of cell apoptosis in rs5951785 near miR-507. **F, G.** Effects of rs5951785 near miR-507 on cell cycle were analyzed with flow cytometry. (G) Results quantitated in cell cycle were shown in histogram. There was no difference of cell cycle between miR-507 wild and miR-507 mutant type. Each data point represented the mean ± SE from three separate experiments in which treatments were performed in triplicate. *P<0.05, **P<0.01, ***P<0.001.

**Figure 3:**
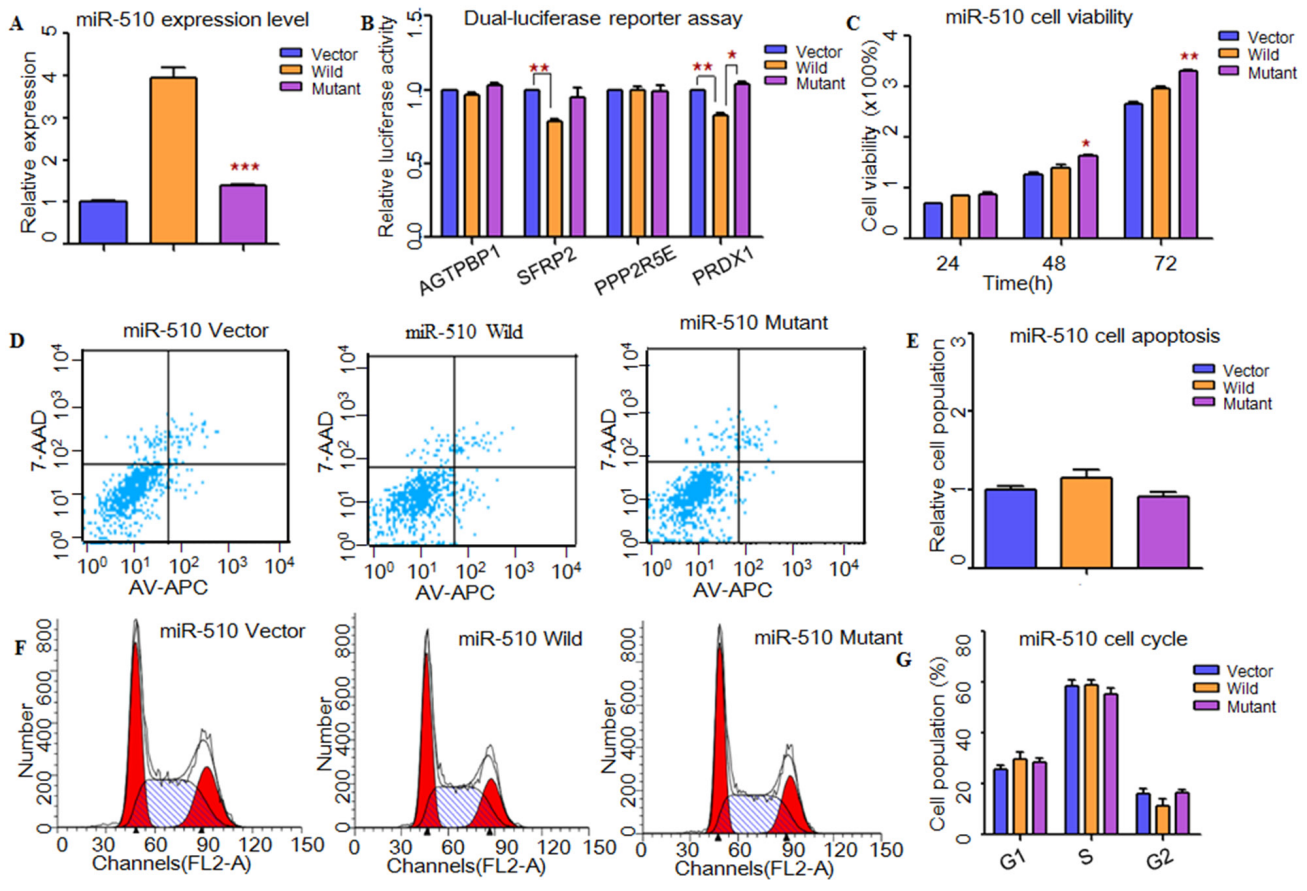
Functional analysis indicated that rs1447393 near miR-510 might protect from the risk of NOA **A.** RT-PCR was applied to detected mature miR-510 expression levels. The miR-510 expression level was significant down-regulated in mutant type compared with the wild type. **B.** Dual-luciferase reporter assay was conducted to measure the effects of rs1447393 near miR-510 on promoter-transcriptional activity. It was significantly increased in miR-510 mutant type for PRDX1. **C.** CCK8 was used to determine the influence of rs1447393 near miR-510 on cell growth. The cell growth was markedly increased with miR-510 mutant type at 48h. **D, E.** Flow cytometry assay was performed to analyze the effects of rs1447393 near miR-510 on cell apoptosis. (E) Histogram of cell apoptosis was presented to depict cell apoptotic percentages. There was no significant difference of cell apoptosis in rs1447393 near miR-510. **F, G.** Effects of rs1447393 near miR-510 on cell cycle were determined by flow cytometry. (G) Results quantitated in cell cycle were shown in histogram. There was no difference of cell cycle between miR-510 wild and miR-510 mutant type. Each data point represented the mean ± SE from three separate experiments in which treatments were performed in triplicate. *P<0.05, **P<0.01, ***P<0.001.

To understand the impacts of these two SNPs (rs5951785 near hsa-miR-506/507; rs1447393 near hsa-miR-510) on the miRNA expression, we transfer the wild-type pre-miRNAs and mutant pre-miRNAs into HEK-293T cells. Through qPCR, we found that the expression levels of these three miRNAs were all significantly down-regulated in mutant type (rs5951785 near hsa-miR-506, *P*=4.47×10^−2^, Figure 1A; rs5951785 near hsa-miR-507, *P*=4.80×10^−3^, Figure 2A; rs1447393 near hsa-miR-510, *P*=3.00×10^−4^, Figure 3A).

Next, we used TargetScan, miRanda and miRwalk to predict the putative targets of these three miRNAs (Supplementary Material, Tables S4-S5). Through conducting dual-luciferase reporter assay, we found that the luciferase activities of *CDK4*, *GLI3*, *PIK3C2A*, *ADAM17, SFRP2* and *PRDX1* were significantly decreased when compared to the vectors, suggesting that they were the potential targets of hsa-miR-506, hsa-miR-507 and hsa-miR-510, respectively (Figure 1B, 2B, 3B). Then, we wanted to know whether these SNPs changed miRNAs' binding ability with its targets. As results shown, the relative luciferase activities were significantly increased with hsa-miR-506 mutant type for *GLI3* (*P*=1.11×10^−5^) and *PIK3C2A* (*P*=4.00×10^−4^, Figure 1B), hsa-miR-507 mutant type for *ADAM17* (*P*=2.00×10^−4^, Figure 2B) and hsa-miR-510 mutant type for *PRDX1* (*P*=2.00×10^−3^, Figure 3B) compared with wild-type, respectively. However, to the *CDK4* (Figure 1B) and *SFRP2* (Figure 3B), no significant difference was observed between the wild-type and mutant-type.

### Effects of MiRNAs and sNPs on cell functions

To investigate whether these X-linked miRNAs involved in cell function, HEK-293T cells were transfected with miRNAs containing wild type and mutant allele. As shown in Figure 1, cell growth was significantly decreased at 48h (*P*=1.00×10^−2^) and 72h (*P*=9.30×10^−3^) with hsa-miR-506 mutant type (Figure 1C). It suggested that rs5951785 near hsa-miR-506 exerted a growth-inhibiting function. The cell growth was markedly increased at 48h (*P*=4.16×10^−2^) and 72h (*P*=4.90×10^−3^) with hsa-miR-510 mutant allele (Figure 3C), indicating that rs1447393 near hsa-miR-510 promoted cell proliferation. No significant difference was observed in the rs5951785 near hsa-miR-507 (Figure 2C). Besides, we also found hsa-miR-506 mutant-type significantly increased cell apoptosis (*P*=1.69×10^−2^, Figure 1D, 1E), while there was no difference in hsa-miR-507 nor hsa-miR-510 between the wild and mutant alleles (Figure 2D, 2E; Figure 3D, 3E), implicating that growth inhibition was accompanied with increased apoptosis population. No significant difference was observed in cell cycle (Figure 1F, 1G; Figure 2F, 2G; Figure 3F, 3G).

## DISCUSSION

X-linked miRNAs are important to spermatogenesis, but till now few studies have investigated the relationship between X-linked miRNAs and male infertility. To comprehensively investigate their relationship, we conducted X chromosome-wide miRNAs capture sequencing and large-scale follow-up genotyping. Our results demonstrated that i) rs5951785 near hsa-miR-506/507 was associated with significantly increased risk of NOA, while rs1447393 near hsa-miR-510 decreased the risk of NOA; ii) rs5951785 significantly decreased binding affinity of hsa-miR-506, inhibited cell proliferation and promoted cell apoptosis.

Several studies have reported that the SNPs in pre-miRNAs could reduce the production of mature miRNAs [16, 17]. Consistent with these related studies, the expression levels of these three miRNAs were all down-regulated with mutant alleles. Through dual luciferase report assays, we found rs5951785 and rs1447393 decreased their binding activity with mRNA 3′UTR of *GLI3, PIK3C2A*, *ADAM17* and *PRDX1*, respectively.

*GLI3*, a potential target of miR-506, has been reported highly expressed in spermatogonia cells (both type A and type B) [18]. It could inhibit cell proliferation and promote cell death [19], which was consistent with our functional analysis of rs5951785 near hsa-miR-506, suggesting that *GLI3* might be associated with NOA. *ADAM17*, a target of miR-507, is involved in germ cell apoptosis during spermatogenesis [20]. Environmental toxicants exposures can also active *ADAM17* to induce the extrinsic pathway of apoptosis in the germ cell [21]. However, we didn't observe apoptosis changes after overexpress hsa-miR-507. This might due to the low expression level of *ADAM17* in HEK-293T cell. *PRDX1*, a target gene of hsa-miR-510, was reported to participate in removing the ROS [22]. Spermatozoa is highly susceptible to ROS-induced damage and dysregulation of *PRDX1* might induce sperm DNA damage.

In conclusion, rs5951785 near hsa-miR-506/507 and rs1447393 near hsa-miR-510 were identified to be potential modifier of NOA. Our findings further highlighted that genetic variations in miRNAs might play important roles in the pathogenesis of NOA and might serve as therapeutic targets for NOA.

## MATERIALS AND METHODS

### Study design and participants

This study was approved by the institutional review board of Nanjing Medical University, China (FWA00001501) and conducted according to the Declaration of Helsinki. All participants were voluntary and completed the informed consent in written before taking part in this research.

A three-stage case-control study was conducted: we captured and sequenced all X-linked miRNAs by customer designed array (Agilent SureSelect DNA Capture Array) in 96 NOA affected individuals and 96 fertile controls in screening stage, and a subsequent 2-stage replication with large-scale follow-up genotyping in large cohorts of NOA-affected subjects and fertile controls was performed. The subjects included in screening stage were recruited from the Nanjing Center of Reproductive Medicine between March 2010 and January 2013. The first replication stage (stage I) included 548 NOA cases recruited from the infertility clinic at the Affiliated Hospitals of Nanjing Medical University at Jiangsu (NJMU Infertile study) and 560 healthy male controls from the same hospitals during the same period. And the second replication stage (stage II) included 465 NOA cases sampled from Renji Hospital, Shanghai, and 537 healthy male controls from the same hospital. Some cohorts within the sample sets have been reported in previously published data [23, 24].

### X-linkd miRNAs sequencing

Through miRNASNP (http://www.bioguo.org/miRNASNP/), we included 91 X-linked miRNAs (Supplementary Material, Table S6) in the study [25]. Customer designed Agilent SureSelect Arrays, which target pre-miRNAs regions and enlonger +/− 1KB, were used to capture DNA by hybridization. The hybridization and enrichment was carried out on AB 2720 Thermal Cycler (Life Technologies, USA). Captured DNA was enriched by SureSelect Target Enrichment Kit (Agilent technologies, USA) and used to construct library by Truseq DNA Sample preparation Kit following the standard protocol (Illumina, USA). Sequencing was performed on the Illumina HiSeq 2000 to generate paired-end 100bp reads. Sequencing data was deposited in NCBI SRA with accession number SRP072055.

### Identifying X-linked miRNA SNVs from sequencing data

After checking quality of sequencing reads by FastQC (http://www.bioinformatics.babraham.ac.uk/projects/fastqc/), low quality, short reads and 3′ end of reads with a quality less than 15 were trimmed by FASTX-Toolkit (http://hannonlab.cshl.edu/fastx_toolkit/index.html). For calling of variants, good quality reads were mapped to the human reference genome (UCSC, hg19) using Burrows-Wheeler Aligner (BWA) [26]. Genome Analysis Toolkit (GATK) was used for realignment, base quality score recalibration and duplicate remove [27]. In brief, VarScan and SAMtools were used to identify variants with in-house parameters [28, 29]. SNPs, In/Dels and mutations were annotated using dbSNP132 or the 1000 Genomes Project.

### Follow-up genotyping of screened genetic variants by SNPscan and SNaPshot

Candidate SNVs were genotyped in additional cases and controls by a custom-by-design 48-Plex SNPscan^™^ Kit (Cat#:G0104; Genesky Biotechnologies Inc., Shanghai, China). This kit was developed according to patented SNP genotyping technology by Genesky Biotechnologies Inc., which was based on double ligation and multiplex fluorescence PCR [30]. In order to validate the genotyping accuracy using SNPscan^™^ Kit, 5% duplicate samples were analyzed by single nucleotide extension using the Multiplex SNaPshot Kit (Applied Biosystems Inc., Foster City, CA, USA), and the concordance rates were more than 99%.

### Statistical analysis

Stata package (Stata Corp, LP) and P-link packages were used for data analyses [31]. Multivariate logistic regression under additive genetic model was used to calculate the odds ratios (ORs) and 95% confidence intervals (CIs) of the associations between SNPs and NOA risks. The Cochrane Q statistics test was used for the assessment of heterogeneity. Mann–Whitney test was used to compare the gene expression levels between different groups.

### Target gene prediction and luciferase reporter assays

TargerScan (http://www.targetscan.org/), miRanda (http://www.microrna.org/microrna/home.do) and miRwalk (http://www.umm.uni-heidelberg.de/apps/zmf/mirwalk/), were applied to identify the potential mRNA targets of the three X-linked miRNAs (Supplementary Material, Tables S4-S5). Luciferase Reporter Assayswas descripted previously [32]. All the sequences of pre-miRNAs were confirmed by Sanger sequencing and shown in the Supplementary Material, Table S7.

### Cell proliferation, cell cycle and cell apoptosis assay

Cells were seeded in 96-well plates. 24, 48 and 72 hours after transfection, CCK8 solution (Vazyme, China) was add to each well, then continued incubating for 1 hour at 37°C. The absorbance of each well was detected using TECAN Infinite M200 multimode microplate reader (Tecan, Belgium). As to cell cycle and apoptosis, cells were collected 24 hours after transfections and measured by FACS (BD, USA). All experiments were performed in triplicate independently.

## SUPPLEMENTARY MATERIALS TABLES




