# Supplementary material for: X chromosome-wide identification of SNVs in microRNA genes and non-obstructive azoospermia risk in Han Chinese population
Source: Oncotarget. 2016 Apr 16;7(31):49122–9. doi: 10.18632/oncotarget.8759 (PMC5226495; doi:10.18632/oncotarget.8759)
Supplement: Supplementary file 1 [file oncotarget-07-49122-s001.pdf]

# X chromosome-wide identification of SNVs in microRNA genes and non-obstructive azoospermia risk in han chinese population

## SUPPLMENTARY TABLES

**Supplementary Table S1: NOA associated SNPs in human X-linked miRNAs identified in screening stage through next generation sequencing (NGS)**

| MiRNA | ACC | Location in X chr. (hg19) | SNP | Nucleotide change | Cases[a] | Controls[a] | OR | *P* value |
|---|---|---|---|---|---|---|---|---|
| hsa-mir-105-2 | MI0000112 | 151562884-151562964 | rs5970293 | G > C | 68/26 | 77/14 | 2.10(1.26-3.52) | 0.004 |
| hsa-mir-1277 | MI0006419 | 117520357-117520434 | rs5956046 | T > C | 47/46 | 32/57 | 0.55(0.36-0.84) | 0.005 |
| hsa-mir-1912 | MI0008333 | 113886019-113886098 | rs12836771 | A > G | 85/7 | 77/14 | 0.45(0.23-0.89) | 0.020 |
| hsa-mir-325 | MI0000824 | 76225829-76225926 | rs5981521 | T > C | 78/16 | 69/24 | 0.59(0.36-0.97) | 0.038 |
| hsa-mir-325 | MI0000824 | 76225829-76225926 | rs5938804 | T > C | 78/16 | 70/24 | 0.60(0.36-0.99) | 0.044 |
| hsa-mir-325 | MI0000824 | 76225829-76225926 | rs958410 | A > G | 74/17 | 60/25 | 0.55(0.34-0.91) | 0.018 |
| hsa-mir-3937 | MI0016593 | 39520470-39520575 | rs5917320 | A > G | 69/20 | 52/30 | 0.50(0.31-0.81) | 0.004 |
| hsa-mir-4329 | MI0015901 | 112023946-112024016 | rs11152741 | G > A | 61/32 | 79/15 | 2.76(1.69-4.53) | 0.00004 |
| hsa-mir-4329 | MI0015901 | 112023946-112024016 | rs2286063 | G > T | 64/30 | 79/15 | 2.47(1.50-4.06) | 0.0003 |
| hsa-mir-4330 | MI0015902 | 150336694-150336798 | rs547043 | A > G | 52/42 | 36/58 | 0.50(0.33-0.76) | 0.0009 |
| hsa-mir-506 | MI0003193 | 146312238-146312361 | rs5951785 | A > G | 77/14 | 87/10 | 1.86(1.02-3.36) | 0.040 |
| hsa-mir-507 | MI0003194 | 146312502-146312595 | rs5905010 | C > G | 75/18 | 84/10 | 2.02(1.12-3.63) | 0.018 |
| hsa-mir-509-3 | MI0005717 | 146341170-146341244 | rs2504174 | C > T | 45/49 | 35/59 | 1.55(1.03-2.34) | 0.037 |
| hsa-mir-510 | MI0003197 | 146353853-146353926 | rs61560773 | G > C | 85/9 | 76/17 | 0.47(0.26-0.87) | 0.015 |
| hsa-mir-510 | MI0003197 | 146353853-146353926 | rs1447394 | G > T | 84/10 | 73/19 | 0.46(0.25-0.82) | 0.008 |
| hsa-mir-510 | MI0003197 | 146353853-146353926 | rs1447393 | C > G | 83/5 | 73/13 | 0.34(0.16-0.73) | 0.004 |
| hsa-mir-510 | MI0003197 | 146353853-146353926 | rs112589319 | T > A | 86/8 | 75/18 | 0.39(0.21-0.73) | 0.002 |
| hsa-mir-652 | MI0003667 | 109298557-109298654 | rs5985440 | A > G | 89/5 | 77/17 | 0.25(0.12-0.53) | 0.0001 |
| hsa-mir-652 | MI0003667 | 109298557-109298654 | rs12841904 | G > A | 88/6 | 78/16 | 0.33(0.17-0.97) | 0.001 |
| hsa-mir-766 | MI0003836 | 118780701-118780811 | rs5909648 | C > A | 87/7 | 80/14 | 0.46(0.23-0.90) | 0.022 |
| hsa-mir-766 | MI0003836 | 118780701-118780811 | rs6646439 | A > T | 87/7 | 80/14 | 0.46(0.23-0.90) | 0.022 |
| hsa-mir-767 | MI0003763 | 151561893-151562001 | rs5970291 | G > A | 68/26 | 77/14 | 1.73(1.06-2.83) | 0.027 |

Chr, chromosome; ACC, Accession number in miRbase;NOA, non-obstructive azoospermia; OR, odds ratio.

[a] Major allele/minor allele.

[b] OR and *P* values were calculated by allele model.

**Supplementary Table S2: Validation of NOA associated SNPs near human X-linked miRNAs in large population (Stage I validation)**

| MiRNA | ACC | Location in X chr. (hg19) | SNP | Nucleotide change | Cases[a] | Controls[a] | OR[b] | *P* value[b] |
|---|---|---|---|---|---|---|---|---|
| hsa-mir-105-2 | MI0000112 | 151562884-151562964 | rs5970293 | G > C | 416/118 | 415/142 | 0.83(0.63-1.10) | 0.188 |
| hsa-mir-1277 | MI0006419 | 117520357-117520434 | rs5956046 | T > C | 343/186 | 350/207 | 0.92(0.72-1.17) | 0.492 |
| hsa-mir-1912 | MI0008333 | 113886019-113886098 | rs12836771 | A > G | 466/72 | 477/79 | 0.93(0.66-1.32) | 0.692 |
| hsa-mir-325 | MI0000824 | 76225829-76225926 | rs5981521 | T > C | 463/73 | 470/90 | 0.82(0.59-1.15) | 0.255 |
| hsa-mir-325 | MI0000824 | 76225829-76225926 | rs5938804 | T > C | 457/74 | 462/88 | 0.85(0.61-1.19) | 0.342 |
| hsa-mir-325 | MI0000824 | 76225829-76225926 | rs958410 | A > G | 452/81 | 448/107 | 0.75(0.55-1.03) | 0.076 |
| hsa-mir-3937 | MI0016593 | 39520470-39520575 | rs5917320 | A > G | 378/151 | 383/176 | 0.87(0.67-1.13) | 0.291 |
| hsa-mir-4329 | MI0015901 | 112023946-112024016 | rs11152741 | G > A | 415/117 | 425/133 | 0.90(0.68-1.20) | 0.470 |
| hsa-mir-4329 | MI0015901 | 112023946-112024016 | rs2286063 | G > T | 415/117 | 427/132 | 0.91(0.69-1.21) | 0.524 |
| hsa-mir-4330 | MI0015902 | 150336694-150336798 | rs547043 | A > G | 225/302 | 277/272 | 1.37(1.07-1.74) | 0.011 |
| hsa-mir-506/507 | MI0003193 | 146312238-146312361 | rs5951785 | A > G | 423/113 | 471/85 | 1.48(1.09-2.02) | 0.013 |
| hsa-mir-507 | MI0003194 | 146312502-146312595 | rs5905010 | C > G | 434/101 | 473/85 | 1.30(0.94-1.78) | 0.109 |
| hsa-mir-509-3 | MI0005717 | 146341170-146341244 | rs2504174 | C > T | 236/292 | 243/314 | 0.96(0.75-1.22) | 0.723 |
| hsa-mir-510 | MI0003197 | 146353853-146353926 | rs61560773 | G > C | 469/59 | 480/80 | 0.75(0.53-1.08) | 0.125 |
| hsa-mir-510 | MI0003197 | 146353853-146353926 | rs1447394 | G > T | 443/77 | 439/93 | 0.82(0.59-1.14) | 0.239 |
| hsa-mir-510 | MI0003197 | 146353853-146353926 | rs1447393 | C > G | 462/47 | 469/89 | 0.54(0.37-0.78) | 0.001 |
| hsa-mir-510 | MI0003197 | 146353853-146353926 | rs112589319 | T > A | 481/53 | 493/66 | 0.82(0.56-1.21) | 0.319 |
| hsa-mir-652 | MI0003667 | 109298557-109298654 | rs5985440 | A > G | 463/29 | 471/54 | 0.55(0.34-0.87) | 0.012 |
| hsa-mir-652 | MI0003667 | 109298557-109298654 | rs12841904 | G > A | 462/74 | 467/91 | 0.82(0.59-1.15) | 0.248 |
| hsa-mir-766 | MI0003836 | 118780701-118780811 | rs5909648 | C > A | 477/57 | 489/69 | 0.85(0.58-1.23) | 0.382 |
| hsa-mir-766 | MI0003836 | 118780701-118780811 | rs6646439 | A > T | 456/61 | 484/69 | 0.94(0.65-1.35) | 0.734 |

Chr, chromosome; ACC, Accession number in miRbase;NOA, non-obstructive azoospermia; OR, odds ratio.
[a] Major allele/minor allele.
[b] OR and P values were calculated by additive model.

**Supplementary Table S3: Validation of NOA associated SNPs near human X-linked miRNAs in large population (Stage II validation)**

| MiRNA | ACC | Location in X chr. (hg19) | SNP | Nucleotide change | Cases[a] | Controls[a] | OR[b] | P value[b] |
|---|---|---|---|---|---|---|---|---|
| hsa-mir-506/507 | MI0003193 | 146312238-146312361 | rs5951785 | A > G | 386/85 | 450/60 | 1.65(1.16-2.36) | 0.006 |
| hsa-mir-510 | MI0003197 | 146353853-146353926 | rs1447393 | C > G | 423/37 | 471/65 | 0.63(0.41-0.97) | 0.035 |
| hsa-mir-652 | MI0003667 | 109298557-109298654 | rs5985440 | A > G | 473/35 | 495/41 | 0.89(0.56-1.43) | 0.637 |

Chr, chromosome; ACC, Accession number in miRbase;NOA, non-obstructive azoospermia; OR, odds ratio.
[a] Major allele/minor allele.
[b] OR and P values were calculated by additive model.

**Supplementary Table S4: Predicted or reported targets of selected human X-miRNAs**

| Selected x-linked miRNA | Predicted targetes | References | Algorithm | | | annotation |
|---|---|---|---|---|---|---|
| | | | Targetscan | miRanda | miRwalk | |
| **MiRNA-506** | *CDK4* | PMID:24811707 | | | | PMID:12754735 *CDK4* was expressed during spermatogenesis. |
| | *CDH2* | PMID:25230372 | | | | PMID:25631347 *CDH2* was associated with spermatogenesis failure. |
| | *GLI3* | PMID:24608427 | | | | PMID:9178901 *Gli3* is expressed during spermatogenesis. |
| | *PIK3C2A* | | $P_{CT}$ >0.99 Context score= -0.6 | mirSVR score= -1.84 | — | PMID:21963845 *PIK3C2A* involved in cell proliferation. |
| | *STAT3* | PMID:24811707 | | | | PMID:24560784 *STAT3* promoted later spermatogonial differentiation steps. |
| **MiRNA-507** | *ADAM17* | | Context score= -0.59 | mirSVR score= -1.61 | — | PMID: 25474107 ADAM17 was associated with male germ cell apoptosis. |
| | *CAMK4* | | Context score= -0.68 | mirSVR score= -2.06 | — | PMID:10932193 *CAMK4* was expressed during spermatogenesis. |
| | *NRF2* | PMID:24307696 | | | | PMID:23089668 *NRF2* associated with sperm motility. |
| **MiRNA-510** | *AGTPBP1* | | | | √ | |
| | *SFRP2* | | Context score= -0.26 | mirSVR score= -1.31 | — | |
| | *PPP2R5E* | | Context score= -0.69 | mirSVR score= -2.32 | — | |
| | *PRDX1* | PMID:23971998 | | | — | PMID:22640168 *PRDX1* associated with sperm motility. |

**Supplementary Table S5: The binding site of human X-linked miRNAs in target genes**

<table>
<tr><td>miR-506</td><td>Position of 131-137 of CDK4 3’UTR<br>hsa-miR-506</td><td>5’ ... CAGAGAUUACUUUGCUGCCUUAA...<br>| |     |||||||<br>3’ AGAUGAGUCUUCCCACGGAAU</td></tr>
<tr><td></td><td>Position of 226-232 of CDH2 3’UTR<br>hsa-miR-506</td><td>5’ ... ACUGGGAUUUUAUGUGCCUUU..<br>|||||||<br>3’ AGAUGAGUCUUCCCACGGAAU</td></tr>
<tr><td></td><td>Position of 1206-1211 of GLI3 3’UTR<br>hsa-miR-506</td><td>5’ ... AGUAGCUGUGUGGAUGCCUUU...<br>||||||<br>3’ AGAUGAGUCUUCCCACGGAAU</td></tr>
<tr><td></td><td>Position of 615-621 of PIK3C2A 3’UTR<br>hsa-miR-506</td><td>5’ ... AUGUUGUAAAUAAUGGUGCCUUA...<br>|||||||<br>3’ AGAUGAGUCUUCCCACGGAAU</td></tr>
<tr><td></td><td>Position of 1208-1214 of STAT3 3’UTR<br>hsa-miR-506</td><td>5’ ... GUUGUUUCUGUGGGUGCCUUA...<br>| |     |||||||||<br>3’ AGAUGAGUCUUCCCACGGAAU</td></tr>
<tr><td>MiR-507</td><td>Position of 16-22 of ADAM17 3’UTR<br>hsa-miR-507</td><td>5’ ...UUCUGACUUAAGUGUGCAAAA...<br>|||||||<br>3’ AAGUGAGGUUUUCCACGUUUU</td></tr>
<tr><td></td><td>Position of 96-104 of CAMK4 3’UTR<br>hsa-miR-507</td><td>5’ ... UUCUGUUUUUGAGGUGCAAAA..<br>|||||||||<br>3’ AAGUGAGGUUUUCCACGUUUU</td></tr>
<tr><td></td><td>Position of 2501-2506 of NRF2 3’UTR<br>hsa-miR-507</td><td>5’ ... GUUUCUGUGUAAGUGUAAAU...<br>||||||<br>3’ AAGUGAGGUUUUCCACGUUUU</td></tr>
<tr><td>MiR-510</td><td>Position of 255-262 of AGTPBP1 3’UTR<br>hsa-miR-510</td><td>5’ ...CCUUUAAAAAAAAAACCUGAGUA...<br>|||||||<br>3’ CACUAACGGUGAGAGGACUCAU</td></tr>
<tr><td></td><td>Position of 161-167 of SFRP2 3’UTR<br>hsa-miR-510</td><td>5’ ...GCAUCCCCAGCAUUUCCUGAGUU...<br>|||     |||||||<br>3’ CACUAACGGUGAGA-- GGACUCAU</td></tr>
<tr><td></td><td>Position of 517-523 of PPP2R5E 3’UTR<br>hsa-miR-510</td><td>5’ ...UCUUUUGCACAUCUUCCUGAGUU...<br>||||     |||||||<br>3’ CACUAACGGUGAGA--- GGACUCAU</td></tr>
<tr><td></td><td>Position of 183-189 of PRDX1 3’UTR<br>hsa-miR-510</td><td>5’ ... CUUUGGUAGGAAUGGCCUGAGUU...<br>|||||||<br>3’CACUAACGGUGAGA-GGACUCAU</td></tr>
</table>

**Supplementary Table S6: The target regions of X-linked miRNAs on the customer-designed DNA capture array**

**(See Supplementary File 1)**

**Supplementary Table S7: The sequences of pre-miRNAs studied in this study.**

| Pre-miRNA name | Sequences of pre-miRNAs |
|---|---|
| pre-miR-506 | GCCACCACCAUCAGCCAUACUAUGUGUAGUGCCUUAUUCAGGAAGGUGUUACUU AAUAGAUUAAUAUUUGUAAGGCACCCUUCUGAGUAGAGUAAUGUGCAACAUGGA CAACAUUUGUGGUGGC |
| pre-miR-507 | GUGCUGUGUGUAGUGCUUCACUUCAAGAAGUGCCAUGCAUGUGUCUAGAAAUAU GUUUUGCACCUUUUGGAGUGAAAUAAUGCACAACAGAUAC |
| pre-miR-510 | GUGGUGUCCUACUCAGGAGAGUGGCAAUCACAUGUAAUUAGGUGUGAUUGAAA CCUCUAAGAGUGGAGUAACAC |
